# Use of Disease-Modifying Treatments in Patients With Sickle Cell Disease

**DOI:** 10.1001/jamanetworkopen.2023.44546

**Published:** 2023-11-22

**Authors:** Terri Victoria Newman, Jingye Yang, Kangho Suh, Charles R. Jonassaint, Sandra L. Kane-Gill, Enrico M. Novelli

**Affiliations:** 1 Department of Pharmacy and Therapeutics, University of Pittsburgh, Pittsburgh, Pennsylvania; 2 Department of Medicine, University of Pittsburgh, Pittsburgh, Pennsylvania; 3 Heart, Lung and Blood Vascular Medicine Institute, University of Pittsburgh, Pittsburgh, Pennsylvania

## Abstract

**Importance:**

Despite hydroxyurea being an established treatment for sickle cell disease (SCD), it remains underused. The recent approval of the disease-modifying treatments (DMTs) l-glutamine, crizanlizumab, and voxelotor underscores the need to understand the uptake of DMTs in the current treatment landscape.

**Objective:**

To explore characteristics that may be associated with DMT use and to describe observed patterns of yearly DMT use from 2014 to 2021.

**Design, Setting, and Participants:**

This cross-sectional study used administrative claims data from Optum’s deidentified Clinformatics Data Mart Database from January 1, 2014, to September 30, 2021, to identify adults and children with SCD. Data were analyzed from August 1, 2022, to August 28, 2023.

**Exposure:**

Use of DMTs.

**Main Outcomes and Measures:**

Patient characteristics across groups with varying patterns of DMT use and yearly patterns of prescription fills for hydroxyurea, crizanlizumab, voxelotor, and l-glutamine.

**Results:**

A total of 5022 beneficiaries with SCD (2081 [41.4%] aged 18-45 years; 2929 [58.3%] female) were included in sample A (144 [2.9%] inconsistent users, 274 [5.5%] incident users, 892 [17.8%] consistent users, and 3712 [73.9%] non-DMT users). Inconsistent users had a higher prevalence of vaso-occlusive crises (mean [SD], 3.7 [4.7]), splenic complications (6 of 144 [4.2%]), pulmonary complications (36 of 144 [25.0%]), kidney disease (21 of 144 [14.6%]), acute chest syndrome (18 of 144 [12.5%]), and health care visits (eg, mean [SD] inpatient visits, 7.0 [10.7]) compared with the other use groups. Non-DMT users had the lowest prevalence of vaso-occlusive crises (mean [SD], 0.8 [2.4]), acute chest syndrome (109 of 3712 [2.9%]), and inpatient (mean [SD], 2.0 [6.6]) and emergency department (mean [SD], 0.7 [3.1]) visits and the highest proportion of adults 65 years and older (593 of 3712 [16.0%]). In sample B (6387 beneficiaries with SCD), hydroxyurea use modestly increased from 428 of 2188 participants (19.6%) in 2014 to 701 of 2880 (24.3%) in 2021. Use of l-glutamine increased briefly but gradually decreased throughout the study period. In 2021, out of 2880 participants, 102 (3.5%) had at least 1 fill for crizanlizumab and 131 (4.6%) had at least 1 fill for voxelotor. Overall, total DMT use increased from 428 of 2188 participants (19.6%) in 2014 to 815 of 2880 patients (28.3%) in 2021.

**Conclusions and Relevance:**

In this cross-sectional analysis of adults and children with SCD, uptake of DMTs remained low from 2014 to 2021, despite the approval of newer therapies. Notable differences in patient characteristics across varied DMT exposure types necessitate further exploration into factors that facilitate DMT use and the creation of strategies to enhance DMT uptake.

## Key Points

**Question:**

How has the uptake of disease-modifying treatments (DMTs) evolved from 2014 to 2021 with the introduction of new drug treatments for sickle cell disease (SCD), and what characteristics can be observed across different treatment use groups?

**Findings:**

In this cross-sectional study, a sample of 5022 adults and children with SCD found that inconsistent DMT users had a higher prevalence of severe SCD complications and more frequent health care visits, whereas non-DMT users were generally older with milder symptoms. In a second sample of 6387 participants, analysis of annual uptake of DMTs found a modest increase in DMT use from 2014 to 2021; notably, nearly 3 of 4 individuals with SCD did not receive any DMTs.

**Meaning:**

These findings suggest that uptake of DMTs in SCD is low; interventions to increase DMT uptake need to consider patient characteristics.

## Teaser

This cross-sectional study describes patient characteristics associated with use of disease-modifying treatments and evaluates the observed annual patterns of individual and composite use among adults and children with sickle cell disease.

## Introduction

Sickle cell disease (SCD) is an inherited red blood cell disorder that affects 100 000 individuals in the US.^1^ Individuals with SCD experience both acute and chronic complications that contribute to extensive morbidity and mortality across their life span.^1^ Current pharmacological management of SCD primarily focuses on disease-modifying treatments (DMTs), as curative therapies are not a viable option for most individuals with SCD. Prior to 2017, hydroxyurea was the only pharmaceutical DMT available for use in SCD. However, recent advancements in pharmacotherapy have led to the approval of 3 new pharmaceutical DMTs: l-glutamine, crizanlizumab, and voxelotor.^1^

Disease-modifying treatments target key components of the pathophysiological processes of SCD responsible for the main clinical manifestations of the disease and play a key role in the prevention, mitigation, and amelioration of SCD complications.^2,3^ Robust evidence supports hydroxyurea’s effectiveness in reducing rates of vaso-occlusive crises (VOCs) by 44%, as well as reducing the rates of mortality, hospitalizations, and other SCD-related complications.^4,5,6,7,8,9^ Although real-world evidence on novel DMTs is still accumulating, evidence from clinical trials and limited real-world analyses demonstrate that newer agents can also improve the clinical course of SCD and reduce VOCs.^10,11,12^

Despite the proven benefits of DMTs in enhancing patient outcomes, they are underused. Stettler et al^13^ discovered that more than 75% of individuals hospitalized or receiving emergency care for SCD pain were not receiving recommended hydroxyurea. Similarly, the Institute for Clinical and Economic Review conducted a survey of 400 patients with SCD and caregivers (in the case of children) to assess the personal and socioeconomic effects of SCD.^12^ The survey revealed that patients and caregivers reported a mean of 13 VOCs annually, with half requiring hospitalization. Despite the high disease burden, less than 50% of patients reported using any DMTs within the previous month, indicating inadequate uptake.

In this unprecedented era characterized by a fast pace of research progress in SCD, treatment options have expanded rapidly, yet data on their use in practice are scarce, and existing literature evaluating DMT use has limitations. First, it often predates novel DMT approvals.^8,14,15^ Second, it focuses on single DMT outcomes without comprehensive evaluation of all 4 pharmaceutical DMTs.^16^ Third, most literature relies on small, geographically limited samples that focus on a single payer (Medicaid) or a single health care site, which limits generalizability.^15,16^

In summary, the existing literature evaluating the use of DMTs does not provide a comprehensive review of the contemporary treatment landscape with respect to treatment types and population characteristics. To address these gaps in the literature, we used a large claims database inclusive of every US region and analyzed patterns of use of all approved pharmaceutical DMTs. We aimed to describe patient characteristics that may be related to DMT use and evaluate the observed patterns of individual and composite DMT use per year.

## Methods

### Study Design

We conducted a retrospective cross-sectional study of patients with SCD enrolled in commercial and Medicare Advantage plans that followed the Strengthening the Reporting of Observational Studies in Epidemiology (STROBE) reporting guideline. This study was approved as exempt from the need for informed consent by the institutional review board at the University of Pittsburgh due to the use of deidentified claims data.

**Figure 1.**
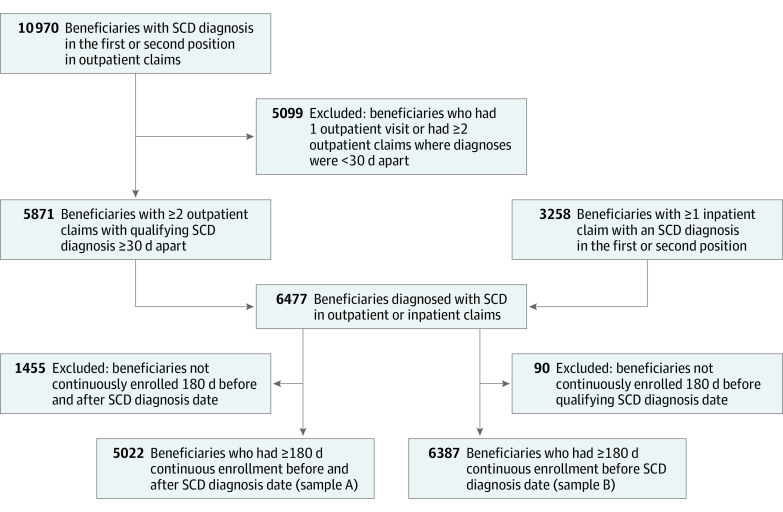
Sample Selection Process Sample A was used to observe baseline characteristics across different disease-modifying treatment (DMT) user groups. Sample B was used to examine DMT use each year from 2014 to 2021. SCD indicates sickle cell disease.

**Figure 2.**
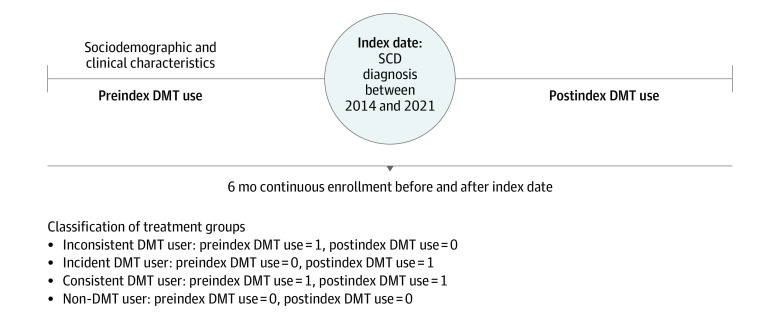
Study Design Process Used to Examine Baseline Characteristics of Different Disease-Modifying Treatment (DMT) Use Groups Within Sample A SCD indicates sickle cell disease.

We used prescription and medical claims data from January 1, 2014, to September 30, 2021, from Optum’s deidentified Clinformatics Data Mart Database (Clinformatics). Our index date was defined as the earliest claim with a qualifying SCD diagnosis (as described in Data Source and Study Population hereinafter) that met our continuous enrollment criteria. Figure 1 shows the process by which we selected our sample population. We identified 2 sample sets and imposed different continuous enrollment criteria to evaluate our 2 aims: to observe baseline characteristics across DMT use groups (sample A; aim 1) and to evaluate individual and total DMT use over time (sample B; aim 2). Figure 2 illustrates the study design process we used to evaluate aim 1. For this analysis, it was necessary for beneficiaries to maintain continuous enrollment for a minimum of 6 months before and after the index date. This 6-month preindex period was used to observe baseline characteristics and evaluate preindex DMT use, and the 6-month postindex period was used to evaluate DMT use and categorize DMT use groups. To evaluate DMT use over time, we required at least 6 months of continuous enrollment before the index date (sample B).

### Data Source and Study Population

We used claims data from Clinformatics, which is a large database that contains administrative health care claims such as inpatient, outpatient, and pharmacy. Covered beneficiaries belong to a large commercial health plan that spans all 50 US states. Figure 1 illustrates the process we used to obtain our 2 samples. To observe characteristics across different DMT use groups, we selected our study sample through the following steps. First, we identified individuals who had an inpatient or outpatient claim with an SCD diagnosis in the first or second position between 2014 and 2021 (lists of *International Classification of Diseases, Ninth Revision* [*ICD-9*] and *International Statistical Classification of Diseases and Related Health Problems*, *Tenth Revision* [*ICD-10*] are given in the eTable in Supplement 1). Second, we included individuals with 1 inpatient or 2 outpatient claims, at least 30 days apart, with an SCD diagnosis in the first or second position, using a validated algorithm.^17^ Third, we included individuals who had at least 6 months of continuous enrollment before and after the index SCD date (sample A; aim 1). To observe DMT use over time, we required at least 6 months of continuous enrollment before the index SCD date (sample B; aim 2).

### Exposure

For aim 1, our primary exposure was DMT use. The DMTs of interest in this study were hydroxyurea, l-glutamine, voxelotor, and crizanlizumab. We categorized 4 treatment groups (Figure 2). Inconsistent DMT users were defined as individuals with at least 1 fill for any DMT in the 6-month preindex period and no fills for any DMT in the 6-month postindex period. Incident DMT users were those with no fills for any DMT in the preindex period and at least 1 fill for any DMT in the postindex period. Consistent DMT users were defined as individuals with at least 1 fill for any DMT in the preindex and postindex periods. Non-DMT users were individuals with no fill for any DMT in the preindex and postindex periods. For aim 2, our exposure was defined by calendar years, spanning from 2014 through 2021, to observe the annual cross-sectional use of DMTs.

### Outcomes

For aim 1, we assessed the baseline characteristics of our total sample population and stratified across our 4 distinct groups of inconsistent, incident, consistent, and non-DMT users. Baseline characteristics included sociodemographic and clinical characteristics and use of health care resources (defined in Covariates subsection of Methods). For aim 2, we evaluated the yearly use of DMTs from 2014 to 2021. We defined yearly use of a DMT as the proportion of individuals with at least 1 fill for the respective DMT among the total amount of individuals with an SCD diagnosis in the corresponding calendar year. To evaluate composite DMT use, we combined the counts of individuals filling prescriptions for any DMT within each year.

### Covariates

Using sample A, we captured baseline characteristics including sociodemographic information, clinical characteristics, medication, and health care utilization. Sociodemographic characteristics included age, sex, and US Census region. We did not include race in our analysis due to limitations in our data source regarding the categorization of race. Clinical characteristics were measured using *ICD-9* and *ICD-10* codes and included SCD complications such as mean number of VOC, stroke, acute chest syndrome, splenic complications, avascular necrosis, pulmonary complications, kidney disease, thrombosis, leg ulcers, and neuropathic pain. Other non–SCD-related clinical characteristics captured included the Charlson Comorbidity Index, hypertension, hyperlipidemia, chronic obstructive pulmonary disease, asthma, congestive heart failure, coronary artery disease, diabetes, anxiety, depression, bipolar disorder, psychosis, schizophrenia, and neoplasms. We also collected information on medication use, including opioids, nonsteroidal anti-inflammatory drugs, acetaminophen, aspirin, antidepressants, and antipsychotic medications. Last, we collected information on inpatient, outpatient, and emergency department health care visits.

### Statistical Analysis

Data were analyzed from August 1, 2022, to August 28, 2023. We conducted descriptive analyses to characterize factors that may be related to DMT use and observed the use of DMTs over time. We used χ^2^, Kruskal-Wallis, and Fisher exact Monte Carlo tests to compare characteristics associated with various treatment groups for quantitative and categorical variables as appropriate. Covariates with 1-sided *P* < .05 for the χ^2^ test and 2-sided *P* < .05 for Fisher exact Monte Carlo test were considered statistically significant. To track changes in DMT use, we calculated annual proportions of individuals with at least 1 fill for a specific DMT (numerator) against those diagnosed with SCD (denominator). This process was repeated for each DMT to observe DMT use for each year. We applied the same mechanism to determine the yearly, overall DMT use, which included the total proportion of individuals who filled any DMT prescription. Analyses were conducted with SAS statistical software, version 9.4 (SAS Institute Inc).

## Results

### Sample Population

A total of 5022 beneficiaries with SCD were included in sample A and 6387 were included in sample B. In sample A, 144 individuals (2.9%) were inconsistent DMT users, 274 (5.5%) were incident DMT users, 892 (17.8%) were consistent DMT users, and 3712 (73.9%) were non-DMT users. In this population, 2081 individuals (41.4%) were aged 18 to 45 years and 1027 (20.5%) were younger than 18 years. A total of 2929 beneficiaries (58.3%) were female and 2093 (41.7%) were male. The mean (SD) 6-month preindex VOC count was 1.3 (3.1).

### Characteristics of DMT Use

The Table shows patient characteristics among our total population (sample A) and stratified by the 4 treatment use groups. Individuals younger than 18 years ranged from 23 of 144 (16.0%) for inconsistent users to 55 of 274 (20.1%) for incident users, 180 of 892 (20.2%) for consistent users, and 769 of 3712 (20.7%) for non-DMT users. Notably, the inconsistent DMT user group had a higher prevalence of individuals aged 18 to 45 years (89 of 144 [61.8%]) compared with incident (151 of 274 [55.1%]), consistent (432 of 892 [48.4%]), or non-DMT (1409 of 3712 [38.0%]) user groups (*P* < .001). In contrast, the non-DMT user group contained a significantly greater proportion of individuals 65 years and older (593 of 3712 [16.0%]) compared with the inconsistent (<10 [1.0%-2.0%]), incident (10 of 274 [3.6%]), and consistent (40 of 892 [4.5%]) users (*P* < .001). The percentage of female patients was notably higher in the inconsistent (86 of 144 [59.7%]) and non-DMT (2217 of 3712 [59.7%]) user groups compared with the incident (146 of 274 [53.3%]) and consistent (480 of 892 [53.8%]) user groups (*P* = .004). However, there were no statistically significant differences in the distribution of US Census regions among treatment use groups.

**Table.** Baseline Characteristics of Sample A

Characteristic	DMT user group^a^					*P* value
	Inconsistent (n = 144)	Incident (n = 274)	Consistent (n = 892)	Non-DMT (n = 3712)	All (N = 5022)	
Age group, y						
<18	23 (16.0)	55 (20.1)	180 (20.2)	769 (20.7)	1027 (20.5)	<.001^b^
18-44	89 (61.8)	151 (55.1)	432 (48.4)	1409 (38.0)	2081 (41.4)	
45-54	18 (12.5)	40 (14.6)	138 (15.5)	538 (14.5)	734 (14.6)	
55-64	12 (8.3)	18 (6.6)	102 (11.4)	403 (10.9)	535 (10.7)	
≥65	<10 (1.0-2.0)	10 (3.6)	40 (4.5)	593 (16.0)	645 (12.8)	
Sex						
Female	86 (59.7)	146 (53.3)	480 (53.8)	2217 (59.7)	2929 (58.3)	.004^c^
Male	58 (40.3)	128 (46.7)	412 (46.2)	1495 (40.3)	2093 (41.7)	
US region						
Midwest	24 (16.7)	51 (18.6)	127 (14.2)	559 (15.1)	761 (15.2)	.08^c^
South	91 (63.2)	175 (63.9)	631 (70.7)	2413 (65.0)	3310 (65.9)	
Northeast	17 (11.8)	26 (9.5)	73 (8.2)	432 (11.6)	548 (10.9)	
West	12 (8.3)	22 (8.0)	58 (6.5)	290 (7.8)	382 (7.6)	
Unknown	<10 (0-2.0)	<10 (0-2.0)	<10 (0-2.0)	18 (0.5)	21 (0.4)	
SCD complications/clinical characteristics						
VOC episode, mean (SD)	3.7 (4.7)	2.2 (4.2)	2.6 (4.2)	0.8 (2.4)	1.3 (3.1)	<.001^d^
Charlson Comorbidity Index, mean (SD)	0.8 (1.2)	0.6 (1.0)	0.7 (1.0)	0.7 (1.2)	0.7 (1.2)	.003^d^
Stroke	<10 (2.0-3.0)	11 (4.0)	30 (3.4)	131 (3.5)	175 (3.5)	.81^b^
Acute chest syndrome	18 (12.5)	22 (8.0)	89 (10.0)	109 (2.9)	238 (4.7)	<.001^c^
Splenic complications	6 (4.2)	<10 (1.0-2.0)	6 (0.7)	35 (0.9)	49 (1.0)	.01^b^
Avascular necrosis	15 (10.4)	19 (6.9)	98 (11.0)	120 (3.2)	252 (5.0)	<.001^c^
Pulmonary complications	36 (25.0)	52 (19.0)	209 (23.4)	579 (15.6)	876 (17.4)	<.001^c^
Kidney disease	21 (14.6)	18 (6.6)	123 (13.8)	394 (10.6)	556 (11.1)	.002^c^
Thrombosis	7 (4.9)	8 (2.9%)	55 (6.2)	115 (3.1)	185 (3.7)	<.001^c^
Leg ulcers	<10 (2.0-3.0)	<10 (1.0-2.0)	26 (2.9)	60 (1.6)	94 (1.9)	.06^b^
COPD or asthma	31 (21.5)	35 (12.8)	173 (19.4)	518 (14.0)	757 (15.1)	<.001^c^
Congestive heart failure	12 (8.3)	16 (5.8)	66 (7.4)	177 (4.8)	271 (5.4)	.01^c^
Coronary artery disease	6 (4.2)	<10 (1.0-2.0)	23 (2.6)	159 (4.3)	192 (3.8)	.01^b^
Diabetes	11 (7.6)	13 (4.7)	58 (6.5)	425 (11.4)	507 (10.1)	<.001^c^
Hypertension	44 (30.6)	53 (19.3)	233 (26.1)	1016 (27.4)	1346 (26.8)	.02^c^
Hyperlipidemia	10 (6.9)	17 (6.2)	70 (7.8)	508 (13.7)	605 (12.0)	<.001^c^
Anxiety	21 (14.6)	19 (6.9)	102 (11.4)	240 (6.5)	382 (7.6)	<.001^c^
Depression	30 (20.8)	21 (7.7)	116 (13.0)	291 (7.8)	458 (9.1)	<.001^c^
Bipolar disorder	<10 (2.0-3.0)	<10 (1.0-2.0)	10 (1.1)	49 (1.3)	66 (1.3)	.42^b^
Psychosis	<10 (0-2.0)	<10 (0-2.0)	<10 (0-2.0)	21 (0.6)	24 (0.5)	.37^b^
Schizophrenia	<10 (0-2.0)	<10 (0-2.0)	<10 (0-2.0)	39 (1.1)	44 (0.9)	.15^b^
Neoplasms	<10 (0-2.0)	<10 (0-2.0)	<10 (0-2.0)	42 (1.1)	49 (1.0)	.34^b^
Neuropathic pain	<10 (2.0-3.0)	<10 (1.0-2.0)	18 (2.0)	95 (2.6)	120 (2.4)	.63^b^
Medication use						
Opioids	111 (77.1)	141 (51.5)	625 (70.1)	1348 (36.3)	2225 (44.3)	<.001^c^
Nonsteroidal anti-inflammatory drugs	31 (21.5)	43 (15.7)	209 (23.4)	507 (13.7)	790 (15.7)	<.001^c^
Aspirin	<10 (0-2.0)	<10 (0-2.0)	<10 (0-2.0)	9 (0.2)	11 (0.2)	.63^b^
Acetaminophen	65 (45.1)	86 (31.4)	389 (43.6)	891 (24.0)	1431 (28.5)	<.001^c^
Antipsychotics	6 (4.2)	6 (2.2)	33 (3.7)	97 (2.6)	142 (2.8)	.22^c^
Antidepressants	26 (18.1)	25 (9.1)	125 (14.0)	329 (8.9)	505 (10.1)	<.001^c^
No. of visits, mean (SD)						
Inpatient	7.0 (10.7)	4.4 (12.6)	4.3 (10.0)	2.0 (6.6)	2.7 (7.9)	<.001^d^
Outpatient	13.3 (18.8)	7.9 (11.5)	11.6 (12.8)	9.1 (14.1)	9.6 (14.0)	<.001^d^
Emergency department	3.5 (9.8)	2.1 (8.6)	1.8 (5.3)	0.7 (3.1)	1.1 (4.4)	<.001^d^

Abbreviations: COPD, chronic obstructive pulmonary disease; DMT, disease-modifying treatments; SCD, sickle cell disease; VOC, vaso-occlusive crisis.

^a^ Unless otherwise indicated, data are expressed as No. (%) of participants.

^b^ Calculated using a Fisher exact Monte Carlo test.

^c^ Calculated using a χ^2^ test.

^d^ Calculated using a Kruskal-Wallis test.

#### SCD Complications

Differences in SCD complications were noted among the treatment use groups. Non-DMT users had the lowest prevalence of acute chest syndrome (109 of 3712 [2.9%]) contrasting with inconsistent DMT users who had the highest prevalence (18 of 144 [12.5%]) (*P* < .001). Vaso-occlusive crises were more prevalent among inconsistent users, who experienced a mean (SD) of 3.7 (4.7) episodes in the preindex period compared with incident (2.2 [4.2]), consistent (2.6 [4.2]), and non-DMT (0.8 [2.4]) users (*P* < .001). Furthermore, inconsistent DMT users had the highest frequency of splenic complications (6 of 144 [4.2%]), pulmonary complications (36 of 144 [25.0%]), and kidney disease (21 of 144 [14.6%]) compared with incident, consistent, and non-DMT users. With respect to avascular necrosis, non-DMT users had the lowest prevalence (120 of 3712 [3.2%]), whereas consistent users had the highest prevalence (98 of 892 [11.0%]) (*P* < .001). Consistent users also had the highest prevalence of thrombosis (55 of 892 [6.2%]) compared with the inconsistent (7 of 144 [4.9%]), incident (8 of 274 [2.9%]), and non-DMT (115 of 3712 [3.1%]) treatment groups (*P* < .001).

#### General Clinical Conditions

Inconsistent users showed a notably higher prevalence of diseases such as chronic obstructive pulmonary disease and/or asthma (31 of 144 [21.5%]), congestive heart failure (12 of 144 [8.3%]), hypertension (44 of 144 [30.6%]), anxiety (21 of 144 [14.6%]), and depression (30 of 144 [20.8%]) compared with incident, consistent, and non-DMT user groups. Non-DMT users had a higher prevalence of diabetes (425 of 3712 [11.4%]) and hyperlipidemia (508 of 3712 [13.7%]) compared with the inconsistent, incident, and consistent user groups. Inconsistent (6 of 144 [4.2%]) and non-DMT (159 of 3712 [4.3%]) user groups had the highest prevalence of coronary artery disease compared with the incident (<10 [1.0%-2.0%]) and consistent (23 of 892 [2.6%]) user groups (*P* = .01). No differences were found in the prevalence of other mental health disorders and neoplasms.

#### Use of Medication and Health Care Services

When observing medication use, differences were found in the use of opioids, nonsteroidal anti-inflammatory drugs (NSAIDs), acetaminophen, and antidepressants across treatment groups. Specifically, inconsistent users had a higher use of opioids (111 of 144 [77.1%]), acetaminophen (65 of 144 [45.1%]), and antidepressants (26 of 144 [18.1%]) compared with the incident, consistent, and non-DMT user groups. In contrast, consistent users were more likely to use NSAIDS (209 of 892 [23.4%]) compared with inconsistent (31 of 144 [21.5%]), incident (43 of 274 [15.7%]), and non-DMT (507 of 3712 [13.7%]) users (*P* < .001). Last, when observing health care utilization, inconsistent DMT users had a higher mean (SD) number of inpatient (7.0 [10.7]), outpatient (13.3 [18.8]), and emergency department (3.5 [9.8]) visits compared with incident, consistent, and non-DMT users.

### DMT Use Over Time

**Figure 3.**
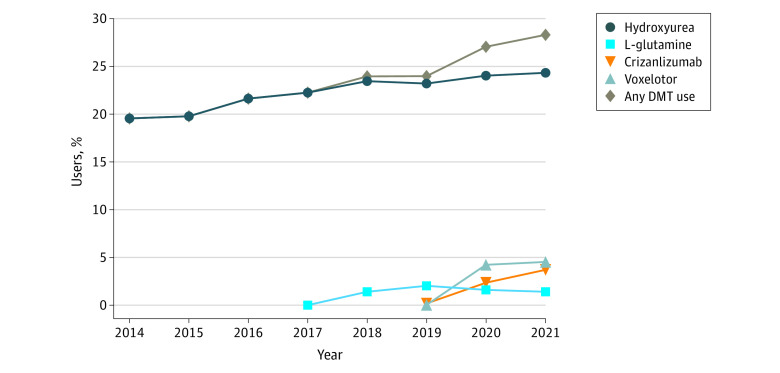
Annual Patterns of Individual and Total Disease-Modifying Treatment (DMT) Use From 2014 to 2021 Claims data were available through September 2021.

Figure 3 shows DMT use from 2014 to 2021 by examining the proportion of DMT users over the total population for each year (sample B). When exploring individual DMT use over the study period, the proportion of hydroxyurea users increased modestly over the 7-year period. In 2014, 428 of 2188 (19.6%) of the total sample population had at least 1 fill for hydroxyurea, which increased to 701 of 2880 (24.3%) in 2021. For l-glutamine, in the years following approval, use reached its peak in 2019 with 70 of 3443 (2.0%) the population having at least 1 fill for the medication. Following 2019, use of l-glutamine decreased. For crizanlizumab, 72 of 3304 (2.2%) of the population had at least 1 fill for the medication in 2020 and 102 of 2880 (3.5%) in 2021. Similarly, with voxelotor, 140 of 3304 (4.2%) of the population had at least 1 fill for the medication in 2020, which slightly increased to 131 of 2880 (4.6%) in 2021. When examining the overall trend in the use of DMTs in SCD between 2014 and 2021, use of DMTs has increased over time, with 428 of 2188 individuals (19.6%) with SCD having at least 1 fill for any DMT in 2014 and 815 of 2880 (28.3%) in 2021.

## Discussion

To our knowledge, this cross-sectional study represents the first comprehensive evaluation of DMT use (inclusive of newer therapies) in a new era of expanded drug treatment options for individuals with SCD. In this study, we descriptively examined characteristics across different DMT use groups and depicted rates of DMT use from 2014 to 2021. In our sample of 5022 patients with SCD, we found that individuals who did not use DMTs were often older adults, female, and relatively healthier, as evidenced by fewer VOC events, severe SCD complications, and hospitalizations. On the other hand, those with various encounter types with DMTs had distinct characteristics compared with non-DMT users. Inconsistent users had a history of more SCD complications and greater use of health care services and opioids. Consistent users also had a history of more SCD complications such as avascular necrosis and thrombosis. Over our study period, from 2014 to 2021, we observed an increase in DMT use within our sample of 6387 individuals. Specifically, 28.3% of individuals had at least 1 prescription fill for a DMT in 2021, an increase from 19.6% in 2014. This overall increase in uptake is mostly due to the availability of new treatment options of voxelotor and crizanlizumab. However, despite an increase over time, use of DMTs was still low, with less than 5% of the population using newer DMTs and less than 25% of the population using hydroxyurea. Most strikingly, nearly 3 of 4 patients were not treated with any DMT, underlining a substantial unmet need for this population.

Limited studies have examined patterns of DMT use on a broad, national level and are reflective of the new treatment landscape. Previous studies have demonstrated suboptimal use of hydroxyurea in populations with SCD.^13,18,19^ Stettler et al^13^ conducted a national analysis of claims data to evaluate hydroxyurea use among patients with a hydroxyurea indication. They found that more than 3 of every 4 patients did not receive treatment. Although we did not evaluate indication for use, these results align with our finding that 2179 patients (75.6%) did not have at least 1 fill for hydroxyurea during the study period. Additionally, our study expands on other studies by evaluating use of DMT in the context of new treatment approvals. We discovered that even with the addition of newer treatments, a considerable number of patients (2065 [71.7%]) did not fill any DMT prescriptions in 2021. To our knowledge, this finding has not been reported previously.

The lack of national awareness and funding for SCD has resulted in limited treatment options for individuals, marked by substandard drug development. In the absence of curative therapies, DMTs can help to prevent and manage SCD complications, leading to favorable effects on health outcomes. Given the demonstrated benefits of DMTs when used appropriately, efforts to increase and optimize DMT use in this patient population are warranted. The most recent guidelines on DMTs were published in 2014 and predate novel therapies.^20^ These guidelines strongly recommend hydroxyurea for the sickle cell anemia genotypes, the most severe and prevalent form of the disease.^20^ While data on hydroxyurea efficacy in non–sickle cell anemia genotypes is limited, the approval of new therapies such as crizanlizumab and voxelotor for all SCD genotypes warrants further studies to guide the use of these agents.

In this study, we discovered that individuals who had some form of DMT use had a higher prevalence of SCD-related complications compared with non-DMT users. Specifically, individuals who had inconsistent or consistent DMT use during the study period had a higher prevalence of SCD complications and use of health care services. While this observation may be due to indication bias, it also emphasizes the importance of exploring barriers to the use of DMTs, including the examination of how disease severity can affect DMT use. Future longitudinal studies should build on this finding to elucidate how different trajectories of DMT use can affect disease outcomes. Additionally, our findings necessitate further evaluation on whether DMTs are initiated reactively or preventively in individuals with SCD. Current treatment guidelines recommend early initiation of hydroxyurea treatment in pediatric patients, regardless of disease severity, to prevent complications. However, recommendations in adults are based on disease severity. As SCD is a chronic, progressive disorder with latent manifestations, preventive therapy is important to reduce acute and chronic events. Thus, these findings highlight the need to further examine the role of DMTs, even among those with less severe disease, to preempt morbidity. Additionally, our study revealed that inconsistent users had the greatest use of health care services and opioids in the preindex period yet had no fills for DMTs in the postindex period. This finding supports previous research indicating that individuals with more severe SCD, who might benefit from DMTs, are not optimally using these treatments.^13^

Our study examined national use of DMT use over the years, capturing the evolving treatment landscape of SCD and depicting characteristics associated with DMT use. This helps address a substantial gap in literature, and future studies should build on our findings. To gain a more comprehensive understanding of DMT use, future analyses should link examinations of use over time with an exploration of the various patient-level, health system–level, and clinician-level factors or barriers that facilitate or impede DMT use. Additionally, as more research becomes available to guide the appropriate use of DMTs, it will be important to conduct further utilization studies to identify areas where intervention strategies can be deployed to improve the use of DMTs.

### Limitations

Our study has limitations. First, our analysis only captures prescription fill data, so we do not have information on actual medication use. This could mean we overestimated DMT use; however, the implications of the findings remain the same: the uptake of DMTs is suboptimal. Second, we did not have access to individual-level factors that may affect eligibility for DMTs. Accounting for these factors would allow us to provide more context to the underuse of DMTs. However, the overarching recommendation is that most of the population with SCD should receive a DMT. Because this study represents a nationally representative population, our findings suggesting that less than 30% of the population receive any form of DMT are an indication that uptake of DMTs is very suboptimal and there is an unmet need for DMTs. Third, Clinformatics data do not include information on patients receiving Medicaid benefits, so our results cannot be extrapolated to this population.

## Conclusions

In this cross-sectional study, we found a modest increase in DMT use from 2014 to 2021 that can be largely attributed to the availability of newer therapies such as crizanlizumab and voxelotor. However, despite these advances, our findings highlight low uptake of DMTs, with nearly 3 of 4 patients not receiving these medications. Additionally, we found that individuals who inconsistently used DMTs had a greater use of health care services and higher prevalence of severe SCD complications, warranting exploration into gaps of DMT use among those with more severe SCD. Our results underscore the importance of conducting additional research to understand and address barriers to DMT utilization. They also highlight the necessity of advocating for clinical and policy initiatives aimed at improving the development and use of DMTs within this population. Ultimately, increasing access to and use of DMTs has the potential to improve outcomes and quality of life for individuals with SCD.

## Notes

Supplement 1. **eTable.** Sickle Cell Disease Diagnosis Codes

Supplement 2.
Data Sharing Statement
