# Supplementary material for: Use of Disease-Modifying Treatments in Patients With Sickle Cell Disease
Source: JAMA Netw Open. 2023 Nov 22;6(11):e2344546. doi: 10.1001/jamanetworkopen.2023.44546 (PMC10665975; doi:10.1001/jamanetworkopen.2023.44546)
Supplement: Supplement 1. — eTable. Sickle Cell Disease Diagnosis Codes [file jamanetwopen-e2344546-s001.pdf]

Supplementary Online Content

Newman TV, Yang J, Suh K, Jonassaint CR, Kane-Gill S, Novelli EM. Use of disease-modifying treatments in patients with sickle cell disease. *JAMA Netw Open*. 2023;6(11):e2344546. doi:10.1001/jamanetworkopen.2023.44546

eTable. Sickle Cell Disease Diagnosis Codes

This supplementary material has been provided by the authors to give readers additional information about their work.

© 2023 Newman TV et al. *JAMA Network Open*.

eTable. Sickle Cell Disease Diagnosis Codes

ICD-9 **Code**	***ICD-10*** **Code**
282.42, 282.62, 282.64, 282.69, 282.41, 282.6, 282.61, 282.63, 282.68	D57.00, D57.01, D57.02, D57.211, D57.212, D57.219, D57.411, D57.412, D57.419, D57.811, D57.812, D57.819, D57.1, D57.20, D57.40, D57.80,

Abbreviations: *ICD= International Classification of Diseases, Ninth Revision [ICD-9] and ICD, Tenth Revision [ICD-10]*

© 2023 Newman TV et al. *JAMA Network Open*.
