# Supplementary material for: Use of Disease-Modifying Treatments in Patients With Sickle Cell Disease
Source: JAMA Netw Open. 2023 Nov 22;6(11):e2344546. doi: 10.1001/jamanetworkopen.2023.44546 (PMC10665975; doi:10.1001/jamanetworkopen.2023.44546)
Supplement: Supplement 2. — Data Sharing Statement [file jamanetwopen-e2344546-s002.pdf]

Data Sharing Statement

Newman. Use of Disease-Modifying Treatments in Patients With Sickle Cell Disease. *JAMA Netw Open*. Published November 22, 2023. doi:10.1001/jamanetworkopen.2023.44546

Data
Data available: No
